# Institutional Treatment of Tuberculosis

**Published:** 1910-11-05

**Authors:** Nathan Raw

**Affiliations:** Physician, Mill Road Infirmary, Liverpool; Member of the International Committee for the Prevention of Tuberculosis.


### Institutional Treatment of Tuberculosis.

By NATHAN RAW, M.D., M.R.C.P. (Lond.), Physician, Mill Road Infirmary, Liverpool; Member of the International Committee for the Prevention of Tuberculosis.

(Read at the First Annual Conference of the British Hospitals Association on September 30, 1910.)

The problem of tuberculosis is a colossal one. It embraces not only the disease itself, but involves the everyday life and surroundings of the poorer classes of the community.

Tuberculosis is to a large extent a house disease, and is greatly influenced in its ravages by poverty and insanitary surroundings. Everything which tends to lower the vitality, such as insanitary and unhealthy surroundings, poor food, alcohol, and debilitating diseases, favours the onset and development of tuberculosis; so that in fighting the disease itself we must also fight against those factors which produce it, and our efforts will only be successful when we have provided the workers with healthy homes and educated them in the knowledge of how to live healthily. The disease chiefly attacks the poor, and is frequently introduced into the larger houses by visitors, nurses, and servants who are suffering from it. When the rich man is attacked with tuberculosis he can take the necessary steps for his cure, and in his absence his family and dependents are comfortably provided for; but when the poor man is attacked he is helpless, and very often has to continue his work until the golden opportunity of a cure has passed away, and he drifts slowly and surely into a state of incurability, until his sufferings are relieved by death.

In the meantime, what happens to his wife and children? They may make a struggle for a time, but in the end often seek the workhouse as a last resource, thus swelling and manufacturing the total of pauperism. It is this utter helplessness and hopelessness when the working classes are attacked with tuberculosis which compels us, as a community, to make some organised effort to stamp out the disease—a disease which has no right to exist in our midst—and which claims as a death-toll every year some 65,000 useful lives, in addition to crippling and deforming thousands more.

The Corporation of Glasgow, in the face of tremendous difficulties, has been in the very forefront in this crusade, and its efforts have been rewarded up to the present by a substantial decrease in tuberculosis; but much yet remains to be done, involving, as it surely will, a great expenditure of thought and money, before this dread disease is stamped out from our life. The loss in wages alone, from those workers afflicted with tuberculosis in Glasgow, is about £250,000 a year, so that from this side of the question alone some great effort should be made to prevent the spread of the disease. It cannot be too strongly emphasised that tuberculosis is always the result of infection, and that it is *never* hereditary.

When the infection is removed no fresh cases will occur, and it can only be by the co-operative action of the health authorities and the people themselves that this great object can be achieved. I would like to direct your attention to-day to the important question of the treatment of tuberculosis in institutions. From the very nature of things it is imperative that a large number of cases must be treated in institutions; the difficult problem to solve is, Who is to be responsible for their treatment? Tuberculosis being an infectious disease, it would naturally follow that the health authorities should deal with it. This might be done in the rural districts, where district councils might combine for the purpose, but in a large city like Glasgow, where it is estimated there are at the present time about 8,000 persons attacked with the disease, the cost would be prohibitive. To provide institutional treatment for 8,000 persons for an indefinite time—in some cases extending over a period of ten years—and to provide for the family in the meantime would be a colossal task for any corporation to undertake. It follows, then, that the work can only be done by a combination of voluntary and rate-aided institutions, and that our chief efforts must be towards prevention. The fact is now well established that the germs of the disease are introduced into the body in a great many cases in early childhood, and I am myself convinced that children infect each other very frequently either in the home or at school. It is absolutely vital to the scheme of prevention that all consumptive children should be removed from the ordinary schools and treated in

### Special Open-Air Schools.

The excellent system of medical inspection of children will disclose all children affected with tuberculosis, and, fortunately, only a small percentage of school children are so affected, so that it is comparatively easy to separate them and educate them in special schools.

### Children's Sanatoria should be provided in a healthy country district for each town, where they could undergo the cure and at the same time receive their education. These sanatoria should be provided by voluntary effort, and if necessary subsidised by a payment from the rates. In all cases, where possible, a portion of the maintenance should be contributed by the parents.

### Treatment of Early Cases.

A great many cases of consumption in the early stages can be cured anywhere, provided they have proper treatment. The best method at present known is the

### Sanatorium.

It is obviously impossible to erect and equip sufficient sanatoria for the treatment of all cases of consumption. I am not an advocate of expensive and luxurious sanatoria; rather would I support the erection of the simple temporary buildings, which would provide the necessary treatment and be capable of renewal every ten years. The educational value of a sanatorium is enormous, and in teaching the consumptive how to live and what precautions to take they almost justify their existence.

### Utilisation of Present Institutions.

All the voluntary hospitals in the country should take their share in the treatment of consumption. At the present time the one disease which causes more deaths than all the rest is rigorously excluded from the voluntary hospitals. Everything is perfectly equipped and ready for the treatment of the sufferers, and all that is required is to adapt a ward or wards to the open-air principles, and an immense amount of good work could be done. I would only advise cases in the early or curative stages for admission; and, in addition to curing the disease, the educational value to students would be very great.

A great many medical students go through their hospital work and never see a case of early consumption, the result being that they are not taught the principles of open-air treatment. This would all be obviated by the admission of such cases to general hospitals. The hospitals of Glasgow could easily find beds for 200 early cases of consumption; and some arrangement might be made with the Corporation to contribute towards their maintenance, in the form of a subsidy or *pro rata*. An arrangement such as this is infinitely preferable to the provision by the City of expensive sanatoria, which means the multiplication of institutions at a heavy initial cost and annual maintenance.

I would like to see all the voluntary hospitals in the country, large and small, set aside a certain percentage of beds for treatment on open-air principles. In many cases the roof could be utilised with good effect, and I have been treating such cases on the balconies of a hospital in the centre of Liverpool for many years with excellent results. With ordinary care there is no danger of infection, and the patients would have the immense advantage of treatment at the hands of skilled physicians and competent nurses.

The advantages would be all-round. The patients would receive the best treatment possible; physicians would have the experience of the disease; students and nurses would be trained in open-air methods; and the advantage to the community would be in the reduction of the death-rate, and the prevention of early cases drifting into chronic and incurable ones, and so becoming chargeable to the rates. The cost could be met by arrangement. The patients themselves should be asked to contribute where possible to their maintenance; the subscribers to the charity could not object, whilst the city, through its rates, would be willing to assist in the work.

### Homes for Advanced Cases.

It is absolutely necessary that some simple and inexpensive institution should be provided where advanced cases could be received until the end. They are the chief means by which the disease is spread to healthy persons, and should be removed from their poor homes, by compulsion if necessary. These institutions might be provided by the health authority. One of the most valuable works performed by the Poor Law has been in the segregation of advanced cases of consumption in the workhouses and infirmaries, so preventing the spread of the disease amongst the poor. In the near future, when matters have been rearranged, this duty will devolve upon the health authorities, as it is no part of the Poor Law system to prevent disease, and when a closer association of the municipalities with the voluntary hospitals is accomplished it will be a simple matter, of easy arrangement, to provide the means of maintenance of such cases in the voluntary hospitals, and at the same time to unite their forces—which at present are disunited—to stamp out a cruel and quite unnecessary disease.

---

### Discussion.

Dr. Mackintosh : I desire one word to point out the difference between Scotland and England once more. Dr. Raw says that he finds cases rigorously excluded, and, as the superintendent of a general hospital, I would like to say that is not the case in Scotland; but this is a matter that we must refer to committee, and not deal with here in detail. I was glad to hear that in Dr. Raw's old hospital in Dundee they are going to have a sanatorium, and Dr. Raw highly approves of that; but he says that the general hospitals in Glasgow could find accommodation for 250. At present they have 300 or 400, principally surgical cases, waiting to get into the general hospital. I want to let Dr. Raw know the condition of affairs now. That might have been possible some years ago, but it is certainly quite impossible now. With regard to utilising the roof, I may mention that in the new Royal Hospital for Sick Children, which is to be erected at Yorkhill, the whole roof is to be used for the children, both day and night; kitchen accommodation and lavatory accommodation are provided. It is the state of things as existing to-day that we have to consider, and not as existing ten or twelve years ago.

### The Work at the Victoria Infirmary.

Dr. Ebenezer Duncan (Victoria Infirmary) : Mr. Chairman, ladies and gentlemen, I would like to emphasise what Dr. Raw has said with regard to voluntary hospitals. I may say that in the Victoria Infirmary, which I represent here, we have two small wards for the treatment of phthisis, on the open-air principle. We have four beds in each. I think that that principle might very properly be increased in the hospitals in Glasgow, and I think it is very necessary that we should isolate these cases of phthisis which occur in the hospitals. We have numerous cases of phthisis sent in for other reasons, surgical and otherwise, and I think it is most important that the suggestion made here—that voluntary hospitals should provide accommodation where these people can be isolated and treated in the open-air—should be taken to heart in every general hospital in Glasgow. Having charge of phthisical wards in the Victoria Infirmary for a great many years, I can say, after my experience there and after my long experience in connection with various sanatoria in the country, I have seen almost as many recoveries in the open wards in the Victoria Infirmary as in the wards of the sanatoria in the working of which I was familiar. I see no reason why this principle should not be developed. The other point that I wish to call attention to is the provision by the Corporation for dying cases of phthisis. For many years that has been urged upon the Corporation, and twenty years ago I read a paper before the Philosophical Society in which I urged the formation of an institution in Glasgow. We know that there are many cases lying in the slums propagating the disease, and these people might, with great advantage to the community, be segregated in an hospital where they would be prevented at any rate from propagating a disease which does so much mischief, and which costs such an enormous sum yearly to the community.

The Need for Segregation.

Dr. Yellowlees (consulting physician, Gartnavel Asylum) : Dr. Duncan has just anticipated what I have felt for a very long time. Where do these cases come from? They certainly come from infection. Where does the infection come from? The patients who are in advanced consumption poison the neighbourhood of their children. Is not that so? If that is so, surely our most urgent duty is to segregate and isolate these cases of infection. I think that is by far the most important thing. I appreciate greatly what is being done for the early consumption cases. The right thing to do is to prevent the early consumption, and I am not speaking without the book, because we are doing that in Glasgow, where we have a hospital for dying consumptives—for those who are so advanced as to be practically, so far as one can see, beyond recovery. The hospital began with eighteen beds, and it will presently have forty-four beds. It must prove a blessing to the poor dying patient, and it must be a great blessing to the family from which the consumptive comes. I should say that the stipulation in such case must be that both lungs have been affected and that the bacilli are present. We are very strict about this; and yet it is very interesting to be able to tell you that, even with those conditions, we have had patients becoming practically well, and of their own accord going out and returning to their employment, showing how certainly consumption, even in its advanced stage, may prove to be recoverable. Of course, the essential thing for the community is to remove from their midst these dangerous centres of infection. It was a great joy to me when Glasgow was able to notify tuberculosis. These cases must be removed from the surroundings where they imperil those whom they are near.

What Corporations can do.

Councillor John M'Pherson (Edinburgh) : Mr. Chairman, ladies and gentlemen, the only reason why I interpose again to make a few remarks is the reference that has been made to Corporations and hospitals dealing with advanced cases. The Corporation of Edinburgh has already, to an extent, solved this difficulty by, some four years ago, resolving to set apart a pavilion at our new City Hospital at Colinton Mains for the purpose of taking in advanced cases. The only thing that would be of importance to this meeting would be a sort of historical gradation of the movement. When the Corporation of Edinburgh resolved to do this thing it had, unfortunately, to deal with a somewhat hostile section of the Press. The criticism was very severe. It was pointed out that this was a mere matter of taking away the people to die, and the consequence was that we had to pass through that criticism at an early stage of the movement. However, the working of the thing has now become well known, and in all the districts of the city there is now a desire to be taken to the hospital, particularly on the part of the families. There are fifty beds in the pavilion, and we have now twelve outside shelters, and in addition to that there are sixty-two beds, and there is rarely one empty for more than a day or two at a time. I have observed as a weakness in the system of dealing with this deadly disease that our medical officer of health can do nothing till a case has reached practically the dying stage. The evil may be going on, and is going on, for weeks and months, but until it can be pronounced as a dying case nothing can be done; and even the mother, in disseminating death to the children, cannot be taken away till she has reached the last stage. Now, to meet that difficulty, we have been able to carry through an arrangement with the Royal Victoria Hospital, whereby we make a considerable contribution to the funds, for which we get the entire use of the dispensary of the Royal Victoria Hospital, and the medical officer of health works along with it. The Royal Infirmary can be used for educational purposes as far as possible, to take away the early cases and keep them there for a week or a month, and educate them into all the principles of self-defence, and then send them back again. We cannot keep them for curative purposes, but we can educate them and take care that they get their friends to adopt the new methods.

The Main Point—Advanced Cases.

Dr. Nathan Raw (in reply) : Mr. Chairman, ladies and gentlemen, at this hour I won't detain you more than a minute, and will only thank you for the discussion which you have given to the paper. I had a sort of suspicion that if I came up to Scotland I would find things already in force, and I am glad to hear that so much has been already done in the big Scotch cities to deal with this problem. Dr. Duncan, in referring to the Victoria Infirmary, says that this is being done on a smaller scale; but what one would like would be that all the voluntary hospitals should make a scheme of treatment and take a larger number of beds. Dr. Yellowlees has hit the nail on the head when he says that the advanced cases are the cause of the disease, and till the advanced cases can be taken out of the small dwellings and insanitary surroundings one cannot but expect that the disease will spread. I hope that the Corporations of every town in the country will see that the advanced cases are removed from their surroundings, and I would suggest that some conference should be held between the voluntary hospitals and the municipal authorities and the Parish Councils, and come and see if some working arrangement could not be arrived at by which the system could be dealt with in every large community. It is only a matter of arrangement, and the municipalities would provide homes for the early cases and would remove the advanced cases from their homes, till the problem is solved through the co-operation of all the authorities. I feel that without that no progress will be made, and when it is done the disease ought to be removed.

Vote of Thanks to the University.

The Chairman : As some of you will have observed, we have the honour to receive a visit from our landlord and good friend, Sir Donald MacAlister, the Principal of the University, and I should like to take this opportunity of asking you to accord our hearty thanks to Sir Donald and the University authorities for their courtesy and kindness in affording us accommodation here where these interesting discussions have taken place. I have heard of a microbe called the *cacoethes scribendi*, but, glancing round these walls, I think the microbe takes the form of the *cacoethes loquendi*. There has been no want of good speaking since this Conference met. I don't want to say anything more about that; but we should like to convey to Sir Donald MacAlister the thanks of this Conference for his kindness in sheltering us here.

Sir Donald MacAlister : Mr. Chairman, ladies and gentlemen, I vindicate the University from having the *cacoethes loquendi*. First of all, I have to apologise for my absence at the commencement of your Conference yesterday, because His Majesty laid upon me certain duties in Belfast; but I came here at the earliest opportunity to show what an interest I take in your Conference. As the head of the University, I have to offer you a cordial welcome, a little late, but none the less sincere. We did the best we could to show our appreciation of your function when we put you in the Humanity Class-room—(laughter)—but I have a further ground for offering you a welcome: that I happen to be the President of the General Medical Council, and I wish to show you how much we who look after one side of the subject appreciate all that is done by the lay workers who give so much of their time—what doctors never can do—to the housekeeping and management of hospitals. I am very glad to see you here and to express my personal obligation for the pains that you have taken to make this Conference a success.

